# Supplementary material for: Dental Caries, Tooth Loss and Quality of Life of Individuals Exposed to Social Risk Factors in Northeast Brazil
Source: Int J Environ Res Public Health. 2023 Aug 28;20(17):6661. doi: 10.3390/ijerph20176661 (PMC10487409; doi:10.3390/ijerph20176661)
Supplement: Supplementary file 1 [file ijerph-20-06661-s001.zip › ijerph-2537394-supplementary.pdf]

Table S1 shows the distribution of individuals according to OHIP-14 dimensions .Considering the potential impact in quality of life indicated by the association of the most negative answers (“often” and “all the time”), it could be observed that physical pain (5.8%) and psychological discomfort (5.8%) were the most frequently occurring OHIP-14 dimensions (Table 2).

Table S1. Distribution of individuals (n= 3063) per frequency of answers classified as “no impact” (never, hardly ever and sometimes), and “impact present” (often and all the time), according to OHIP-14 dimensions.

OHIP-14 dimensions	No impact n (%)	Impact present n (%)
Functional Limitation	3025 (98.8)	38 (1.2)
Physical Pain	2886 (94.2)	177 (5.8)
Psychological Discomfort	2885 (94.2)	178 (5.8)
Physical Disability	2972 (97.0)	91 (3.0)
Psychological Disability	3000 (97.9)	63 (2.1)
Social Disability	3027 (98.8)	36 (1.2)
Handicap	3028 (98.9)	35 (1.1)
